# Positive Rates and Factors Associated with Abnormal Lung Function of Greenhouse Workers in China: A Cross-Sectional Study

**DOI:** 10.3390/ijerph14090956

**Published:** 2017-08-24

**Authors:** Xiaojun Zhu, Panjun Gao, Yishuo Gu, Pei Xiao, Mengxuan Liu, Juan Chen, Yacai Cen, Wenjun Ma, Tao Li

**Affiliations:** 1 National Institute of Occupational Health and Poison Control, Chinese Center for Disease Control and Prevention, Beijing 100050, China; zhuxj@niohp.chinacdc.cn (X.Z.); woodyco@sina.com (P.X.); liumengxuan@niohp.chinacdc.cn (M.L.); 2 Department of Occupational and Environmental Health Sciences, School of Public Health, Peking University, Beijing 100080, China; gaopj@bjmu.edu.cn (P.G.); guyishuo@bjmu.edu.cn (Y.G.); chenjuan94@bjmu.edu.cn (J.C.); 13718507558@163.com (Y.C.)

**Keywords:** greenhouse working, lung function, small airway function

## Abstract

Since the number of greenhouse workers are increasing in China, this observational cross-sectional study was designed to evaluate lung function and discuss the potential risk factors, to provide evidence in the surveillance of greenhouse workers’ health. 678 greenhouse workers in Gansu Province, China were enrolled. A questionnaire which included demographic and occupational information was used. Vital capacity (VC), forced vital capacity (FVC), forced expiratory volume in 1 s (FEV_1_), and FEV_1_:FVC ratios (FEV_1_/FVC), maximal expiratory flow after 50% of the FVC has not been exhaled (MEF_50_), maximal expiratory flow after 25% of the FVC has not been exhaled (MEF_25_) and maximal mid-expiratory flow curve (MMEF) were measured as lung function indicators. The mean values and standard deviations (SDs) of VC% predicted, FVC% predicted, FEV_1_% predicted and FEV_1_/FVC ratio were 106.07 ± 13.36, 107.60 ± 13.95, 97.19 ± 14.80 and 89.76 ± 10.78 respectively. The positive rates of above four and abnormal lung ventilation function were 2.9%, 2.8%, 11.2%, 4.6% and 6.5% respectively. Gender, age, BMI and number of greenhouses owned were influence factors of lung ventilation function (*p* < 0.05). The mean values and SDs of MEF_50_% predicted, MEF_25_% predicted and MMEF% predicted were 69.63 ± 24.95, 54.04 ± 24.94 and 66.81 ± 24.53. The positive rates of above three and abnormal small airway function were 45.0%, 72.1%, 47.2% and 49.4% respectively. Age, education and number of greenhouses owned were influence factors for small airway function (*p* < 0.05). Working in a greenhouse might influence lung function of the workers. Small airway function indicators could be used as priority indicators for the surveillance of greenhouse workers’ health.

## 1. Introduction

Greenhouses have played an important role in agriculture. China has become a country which hosts a huge plant area inside greenhouses, and a large amount of people perform greenhouse work. However, the health conditions of greenhouse workers were not as satisfactory as the economic interests that greenhouse brought. The micro environment inside a greenhouse is semi-closed, thus factors including physical factors, ergonomic factors, chemical factors and biological factors could do harm to greenhouse workers’ health. Physical factors include high temperature and humidity, lack of fresh air, and so on. Ergonomic factors include improper working tools and postures. Chemical factors include pesticide, fertilizer and ethylene, and chlorine gas from greenhouse film. Biological factors mainly include allergens from crops and microorganisms like bacterium, fungi and mites, and so on. 

Previous studies have shown greenhouse workers had high positive rates and increased risk of ocular and skin symptoms [1], bone and joint pain [2], respiratory damage [3] and reproductive disorders including prolonged time-to-pregnancy and spontaneous abortion [4]. In addition, the exposure to pesticide can cause functional injuries to many organs and systems [5,6,7,8].

The respiratory health of greenhouse workers was not satisfactory. Based on related research [3,9], high positive rates and high risk of chronic cough, phlegm, sleep dyspnea, shortness of breath, tightness of chest, chronic bronchitis, asthma and chronic obstructive lung disease (COPD) were found. Since the number of greenhouse workers and positive rates of respiratory adverse health effects is increasing, it is necessary to investigate and monitor their health conditions. Despite to what was reported by Liu S. [9], studies on the respiratory health of greenhouse workers in China were insufficient. Even more, lung function, an important and sensitive index reflecting respiratory health, was rarely tested in the research among greenhouse workers. Therefore, we designed this study to take lung function tests in greenhouse workers in order to research the respiratory health conditions and provide evidence in the surveillance of greenhouse workers’ health. 

## 2. Materials and Methods

### 2.1. Study Design and Subjects

This cross-sectional study was undertaken in Gansu Province, in northwestern China. This area was selected because: (1) most of the adult residents worked as greenhouse farmers; and (2) clinical examination could be performed along with the annual checkup program. The inclusion criteria for all the subjects included: (1) local residents for 1 year and above; (2) age from 18 years and above; (3) working in greenhouse; (4) not pregnant; and (5) properly able to take the lung function test. All the participants read and signed the informed consent. The study was conducted in accordance with the Declaration of Helsinki, and the protocol was approved by the Medical Ethics Committee of the National Institute of Occupational Health and Poison Control, Chinese Center for Disease Control and Prevention, Beijing, China (record number NIOHP201603, 1 August, 2016).

### 2.2. Questionnaires

All the participants voluntarily joined this study with informed consent and then answered a questionnaire conducted by well-trained investigators face to face. The questionnaire asked for information including their demographic information, smoking status, number of greenhouses owned, greenhouse working lifetime, species of crops they planted, whether they wore a mask while working in the greenhouse. Number of greenhouses owned and greenhouse working lifetime were used as the indicators of greenhouse working exposure. 

### 2.3. Lung Function Test

Lung function tests were performed among subjects by using Carefusion^TM^ MasterScreen Pneumo (CareFusion, San Diego, CA, USA, ERS/ATS) according to its instruction manual, after age and height of subjects were measured. Lung ventilation function parameters considered in this study were vital capacity (VC), forced expiratory volume in 1 s (FEV_1_), forced vital capacity (FVC) and FEV_1_:FVC ratios (FEV_1_/FVC). Maximal expiratory flow after 50% of the FVC has not been exhaled (MEF_50_), maximal expiratory flow after 25% of the FVC has not been exhaled (MEF_25_) and maximal mid-expiratory flow curve (MMEF) were considered as indicators of small airway function. All the indicators were shown and analyzed as the percentage of the measured value on the predicted value. Individual predicted values of these indicators were calculated with equations developed by the European Respiratory Society (ERS) in 1993 (equations were showed in Appendix A, Table A1). The ratio of measured value and predicted value that was less than 80% was predicated as reduced index. When VC% and FVC% were less than 80%, it was considered as restrictive ventilation dysfunction in lung. When FEV_1_% were less than 80% or FEV_1_/FVC was less than 70%, it was considered as obstructive ventilation function disturbance of the airways. Only if at least two of MEF_25_%, MEF_50_% and MMEF% were less than 65% could it be predicated as abnormal small airway function.

### 2.4. Statistical Analysis

The data from the questionnaire and clinical examination was collected by using Epidata 3.1 (The EpiData Association, Odense, Denmark). All statistical analysis was performed using SPSS 19 (IBM, Armonk, NY, USA). All the participants were grouped into senior group (age > 47 years) and junior group (age ≤ 47 years) based on the median of age, 4 subgroups (0–5 years, 6–10 years, 11–20 years and >20 years) by greenhouse working lifetime and 3 subgroups (<18.5, 18.5–23.9 and >23.9) by BMI according to the standard of Chinese. The mean values of lung function indicators and the positive rates of abnormal lung function were compared within subgroups of gender, age, BMI, education, status of smoking, number of greenhouses owned, working lifetime and mask using. *T*-test and one-way analysis of variance were used to analyze the differences of mean values in subgroups. Chi-square test and linear trend test were used to analyze the differences of positive rates within subgroups. Logistic regression analysis (backward (LR)) was used to analyze the influence factors. The entry probability for stepwise was 0.05 and the removal probability was 0.10. *p* value of less than 0.05 was considered to indicate statistical significance. Variable assignment and dummy variable contrast coding of multivariate analysis were showed in Appendix A (Table A2 and Table A3). 

## 3. Results 

### 3.1. General Information

A total of 783 participants were investigated, but some of them were excluded because they failed the lung function test (4.7%) or were not qualified according the inclusion criteria or their questionnaires were lack of essential information. Therefore, 678 subject were included. There were 291 (42.9%) male and 387 (57.1%) female. Their mean value and SD of age was 47.51 ± 9.07 years, ranging 24–68 years. The mean value and SD of BMI was 23.78 ± 3.49. The mean value and SD of greenhouse working lifetime was 12.77 ± 6.59 years. 86.6% of the subjects ended their education after middle school or before. 33% were ever-smokers. 89.7% owned no more than 3 greenhouses. 95.0% planted chili as cash crops. 73.3% did not wear a mask while working in the greenhouse (Table 1).

### 3.2. Lung Function

#### 3.2.1. Lung Ventilation Function

##### The Mean Values and SDs of Lung Ventilation Function indicators

The mean values and standard deviations (SDs) of VC% predicted, FVC% predicted, FEV_1_% predicted and FEV_1_/FVC ratio was 106.07 ± 13.36, 107.60 ± 13.95, 97.19 ± 14.80 and 89.76 ± 10.78 respectively. Significant differences of VC% predicted, FVC% predicted, FEV_1_% predicted were observed in the subgroups of gender, BMI, status of smoking, working lifetime (*p* < 0.05). Also, significant uptrends were observed within the subgroups of BMI and working lifetime (*p* < 0.05). Statistical difference of VC% predicted was observed in the subgroups of mask using (*p* < 0.05) (Table 1).

##### Positive Rates of Abnormal Lung Ventilation Function

The positive rates of VC% predicted, FVC% predicted, FEV_1_% predicted, FEV_1_/FVC ratio and abnormal lung ventilation function were 2.9%, 2.8%, 4.6% and 6.5%. The positive rates of FEV_1_% predicted were different between male and female significantly (*p* < 0.05). The positive rates of FVC% predicted and FEV_1_% predicted were statistically different between senior and junior groups (*p* < 0.05). Significantly different positive rates of FEV_1_% predicted were observed in BMI and status of smoking subgroups (*p* < 0.05). The positive rates of VC% predicted and FVC% predicted were different statistically in the subgroups of number of greenhouses owned (*p* < 0.05). Significant downtrend of the positive rate of FVC% predicted was observed in the working lifetime subgroups (*p*_trend_ < 0.05) (Table 2).

##### Influential Factors of Lung Ventilation Function

According to the results of logistic regression, VC% predicted and FVC% predicted of the subjects who had 1 greenhouse were more likely to be abnormal than those who had 2. For FEV_1_% predicted, being male and getting older were risk factors. And FEV_1_% predicted of the subjects whose BMI was over 18.5 were more likely to be abnormal (Table 3).

#### 3.2.2. Small Airway Function

##### The Mean Values and SDs of Small Airway Function Indicators

The mean values and SDs of MEF_50_% predicted, MEF_25_% predicted and MMEF% predicted was 69.63 ± 24.95, 54.04 ± 24.94 and 66.81 ± 24.53. The mean values and SDs of MMEF% predicted were significantly different in age subgroups (*p* < 0.05). The mean values and SDs of MEF_50_% predicted were significantly different in education subgroups (*p* < 0.05). Both MEF_50_% and MMEF% showed significantly decrease trends with the level of education (*p* < 0.05) (Table 4).

##### Positive Rates of Abnormal Small Airway Function

The positive rates of small airway function indicators in total were high. As Table 5 showed, the positive rates of MEF_50_% predicted, MEF_25_% predicted, MMEF% predicted and abnormal small airway function were 45.0%, 72.1%, 47.2% and 49.4% respectively. But significantly different positive rates were only observed in MMEF% predicted and abnormal small airway function in age subgroup (*p* > 0.05).

##### Influential Factors of Small Airway Function

According to the results of binary logistic regression, MEF_25_% predicted of the subjects who had 2 greenhouses were more likely to be abnormal than those who had 1. Age was a risk factor of abnormal MMEF% predicted and small airway function. And MMEF% predicted of the subjects who were educated until middle school were more likely to be abnormal than those who were educated until primary school (Table 6).

## 4. Discussion

Symptoms of respiratory diseases may be silent at the prodromal stage because of the compensatory reaction. Lung function test, as an objective evaluation index, is important and meaningful for the early diagnoses, the determination of lesions’ location, and the identification of the reasons for the shortness of breath. Usually, VC is tested as an index of lung volume. Factors which influence rib cage and the structure of lung tissues will decrease the level of VC. FVC is an indicator to detect restrictive ventilation dysfunction in lung. FEV_1_ and the ratio of FEV_1_ and FVC can reflect obstructive ventilation function disturbance of the airways. VC, FVC, FEV_1_ and FEV_1_/FVC have been used as indicators of respiratory health among agricultural population. Zuskin E. [3] took lung function test in their study related to greenhouse workers. It showed that the level of MEF_75_% predicted in greenhouse workers for over 10 years was significantly lower than that in greenhouse workers for less than 10 years. This result indicated that there might be obstruction in the large airways of greenhouse workers. Also, according to related studies, endotoxin exposure could cause lung functional decline in farmers. Peter F. J. et al. [10] followed 171 pig farmers over 3 years and determined the average exposure to dust and endotoxin in summer and winter to find decline in FEV_1_ and FVC and the correlation between annual decline in FEV_1_ and endotoxin exposure. Heederik D. et al. [11] found a significant relationship between endotoxin exposure and FEV_1_ and a negative relationship between endotoxin concentration and FVC. However, in our study, it was found that positive rates of abnormal lung obstructive ventilation function of greenhouse workers declined by increasing working lifetime. It might be because long term and strenuous physical work enhanced the lung obstructive ventilation function of greenhouse workers. MEF_50_, MEF_25_ and MMEF were used as indicators of small airway ventilation function. MEF_50_ is an index of mid-expiratory flow and MEF_25_ is an index of late expiratory flow. Decreased MMEF can suggest obstruction of small airway, and it is a sensitive index of small airway lesions. The thickening of small airway wall and the destruction of alveolar wall could cause small airway dysfunction. The changes of FEV_1_%, FEV_1_/VC, MEF_50_, MEF_25_ and MMEF reflecting obstructive lung function effect in this study only showed statistical significance, and their practical application value in clinic needs further observation and research. Smoking and respiratory diseases like COPD had influence on small airway function. Cathelicidin (LL-27) is an endogenous antimicrobial peptide, which has antimicrobial activity [12,13], anti-endotoxin activity [14] and chemotaxis [15]. Sun C. et al. [16] examined the expression of LL-37 in small airways from smokers with COPD, smokers without COPD and never-smokers without COPD. Their results showed compared with subjects without COPD, LL-37 immunoreactivity in small airway epithelium was statistically elevated in smokers with COPD. And there was a positive correlation found between the magnitude of LL-37 expression in epithelium and airway wall thickness and collagen deposition. All these results suggested that smoking might influence small airway function through increasing the expression of LL-37 which might stimulate the production of collagen in the underlying lung fibroblasts and make a contribution to small airway remodeling in COPD after smoking exposure.

Eurling I. M. et al. [17] stained and detected the contents of elastin, collagen and hyaluronan of lung tissue sections from smokers, GOLD II and GOLD IV patients. It was found that elastin was notably decreased while collagen and hyaluronan increased in both alveolar and small airway walls among COPD patients. Collagen content in the alveolar wall increased in GOLD IV patients. The value of Elastin dividing over collagen-hyaluronan was used as an index of remodeling. This index decreased more in GOLD II patients than GOLD IV patients, which suggested that matrix component changes were involved in progress airflow limitation and the alterations of alveolar and small airway walls already occurred in moderate COPD.

Our study showed subjects who owned 2 greenhouses were more likely to suffer from small airway dysfunction than those who owned 1 greenhouse. It suggested that more exposure to greenhouse environment might cause the decline of small airway function. Smoking and exposure to other factors like endotoxin and dust [18] could cause inflammatory responses, the matrix components alteration, small airway wall thickening and alveolar wall destruction and thus cause the impairment of small airway function. 

The relationships between greenhouse workers’ lung ventilation function and other factors such as smoking habit, gender and BMI were also evaluated in this study. According to Table 2, smoking may have adverse effect on lung obstructive ventilation function because of the significantly increased of FEV_1_% predicted <80% positive rates in ever-smokers. While the positive rate of FEV_1_/FVC <70% slightly increased in ever-smokers and the difference was not significant. This phenomenon suggested that smoking might reduce greenhouse workers’ lung ventilation function, but perhaps to a lesser extent. This study showed that male greenhouse workers were more likely to suffer from lung ventilation dysfunction, especially obstructive ventilation function disturbance of the airways. The possible reason is that most of smokers are males and smoking may have harmful effects as discussed above. As shown in Table 1, VC and FVC were higher than normal value in all BMI subgroups expect for BMI < 18.5 group. Previous studies showed that FVC% had positive relationship with BMI in children while had negative association with BMI in normal and overweight adults [19,20,21]. Low level of lung function detected in this study may be attributed to malnutrition during childhood and the consequently low BMI at the adult stage. Use of a mask was also investigated, while no evidence that mask use could prevent lung function loss had been detected. Furthermore, there were no significant associations between mask use and other factors such as education, BMI and number of greenhouses owned (Table A4), which indicated that current type and method of mask use cannot provide protection for greenhouse workers in this study. 

There were some limitations in our study. First, our study did not investigate the lung function of controls. Second, the levels of exposure in the greenhouse working environment were not measured, which was because the period when we did the research was after their harvest and the greenhouses were totally open. Therefore, we indicated the occupational exposure with working lifetime and numbers of greenhouses owned. Third, species of crops were unitary because chili was the only main cash crop in the area where we conducted this research. Considering these limitations and the importance of this work in occupational health in China, further studies are necessary.

## 5. Conclusions

Our study found that mild alterations were present in greenhouse workers’ lung ventilation function. High positive rates of abnormal small airway function were detected in greenhouse workers. Greenhouse work might have an influence on the respiratory health of greenhouse workers. Small airway function indicators could be used as priority indicators for the surveillance of greenhouse workers’ health.

## Figures and Tables

**Table 1:** The mean values and standard deviations (SDs) of lung ventilation function indicators among greenhouse workers.

	N (%)	VC% Predicted	FVC% Predicted	FEV_1_% Predicted	FEV_1_/FVC
Total	678 (100)	106.07 ± 13.36	107.60 ± 13.95	97.19 ± 14.80	89.76 ± 10.78
Gender					
Male	291 (42.9)	102.04 ± 12.83 a	104.66 ± 13.89 a	93.86 ± 15.27 a	89.67 ± 10.92
Female	387 (57.1)	109.10 ± 12.95	109.81 ± 13.60	99.69 ± 13.94	89.83 ± 10.69
Age (years)					
≤47	365 (53.8)	105.31 ± 11.96	106.78 ± 12.17	97.94 ± 12.49	90.10 ± 10.76
>47	313 (46.2)	106.95 ± 14.79	108.55 ± 15.75	96.30 ± 17.09	89.36 ± 10.80
BMI					
<18.5	31 (4.6)	98.85 ± 11.87 a,b	99.95 ± 13.68 a,b	89.96 ± 16.37 a,b	86.80 ± 90.17
18.5–23.9	336 (49.6)	106.35 ± 13.27	108.07 ± 14.01	98.07 ± 14.66	90.17 ± 10.57
>23.9	311 (45.9)	106.49 ± 13.42	107.85 ± 13.74	96.95 ± 14.63	89.62 ± 10.90
Education					
Primary school and below	268 (39.5)	107.45 ± 14.84	108.72 ± 15.38	97.74 ± 16.41	90.40 ± 10.04
Middle school	319 (47.1)	105.34 ± 12.24	106.90 ± 13.05	97.45 ± 13.04	89.59 ± 10.99
High school and above	91 (13.4)	104.57 ± 12.21	106.74 ± 12.42	94.61 ± 15.49	88.48 ± 12.05
Smoking					
Never-smokers	454 (67)	107.71 ± 13.16 a	108.75 ± 13.70 a	98.90 ± 14.15 a	89.64 ± 11.24
Ever-smokers	224 (33)	102.75 ± 13.15	105.26 ± 14.19	93.71 ± 15.50	89.82 ± 10.56
Number of greenhouses owned					
1	184 (27.1)	106.07 ± 14.72	107.46 ± 15.27	97.50 ± 16.39	90.55 ± 1050
2	281 (41.4)	106.06 ± 13.02	107.54 ± 13.77	96.62 ± 14.64	89.52 ± 10.49
3	143 (21.1)	106.96 ± 12.31	108.72 ± 12.51	97.92 ± 14.11	89.90 ± 11.84
>3	70 (10.3)	104.32 ± 13.09	105.90 ± 13.94	97.02 ± 12.42	88.40 ± 10.39
Working lifetime (years)					
0–5	104 (15.3)	102.82 ± 12.29 a,b	103.68 ± 13.37 a,b	95.10 ± 14.86 a,b	89.12 ± 11.23
6–10	218 (32.2)	106.56 ± 13.45	108.05 ± 14.19	98.65 ± 14.60	89.21 ± 11.90
11–20	317 (46.8)	106.46 ± 13.21	108.15 ± 13.55	96.98 ± 14.66	90.01 ± 9.97
>20	39 (5.8)	108.88 ± 15.61	111.02 ± 15.74	96.20 ± 16.55	92.50 ± 9.03
Mask using					
No	497 (73.3)	105.41 ± 13.69 a	107.14 ± 14.33	96.58 ± 14.75	89.59 ± 11.26
Yes	181 (26.7)	107.89 ± 12.24	108.87 ± 12.80	98.84 ± 14.85	± 9.33

a: *p* < 0.05; b: *p*_trend_ < 0.05.

**Table 2:** Positive rates of abnormal lung ventilation function among greenhouse workers.

	N (%)	VC% Predicted <80%	FVC% Predicted <80%	FEV_1_% Predicted <80%	FEV_1_/FVC <70%	Abnormal Lung Ventilation Function
Total	678 (100)	20 (2.9)	19 (2.8)	76 (11.2)	31 (4.6)	44 (6.5)
Gender						
Male	291 (42.9)	11 (3.8)	10 (3.4)	43 (14.8) a	17 (5.8)	23 (7.9)
Female	387 (57.1)	9 (2.3)	9 (2.3)	33 (8.5)	14 (3.6)	21 (5.4)
Age (years)						
≤47	365 (53.8)	8 (2.2)	6 (1.6) a	26 (7.1) a	15 (4.1)	21 (5.8)
>47	313 (46.2)	12 (3.8)	13 (4.2)	50 (16.0)	16 (5.1)	23 (7.3)
BMI			s			
<18.5	31 (4.6)	1 (3.2)	3 (9.7)	8 (25.8) a	3 (9.7)	3 (9.7)
18.5–23.9	336 (49.6)	10 (3.0)	8 (2.4)	37 (11.0)	11 (3.3)	17 (5.1)
>23.9	311 (45.9)	9 (2.9)	8 (2.6)	31 (10.0)	17 (5.5)	24 (7.7)
Education						
Primary school and below	268 (39.5)	10 (3.7)	10 (3.7)	33 (12.3)	10 (3.7)	18 (6.7)
Middle school	319 (47.1)	9 (2.8)	8 (2.5)	29 (9.1)	15 (4.7)	19 (6.0)
High school and above	91 (13.4)	1 (1.1)	1 (1.1)	14 (15.4)	6 (6.6)	7 (7.7)
Smoking						
Never-smokers	454 (67)	12 (2.6)	13 (2.9)	43 (9.5)a	18 (4.0)	28 (6.2)
Ever-smokers	224 (33)	8 (3.6)	6 (2.7)	33 (14.7)	13 (5.8)	16 (7.1)
Number of greenhouses owned						
1	184 (27.1)	11 (6.0) a,b	10 (5.4) a,b	21 (11.4)	8 (4.3)	17 (9.2)
2	281 (41.4)	5 (1.8)	5 (1.8)	37 (13.2)	13 (4.6)	15 (5.3)
3	143 (21.1)	2 (1.4)	1 (0.7)	12 (8.4)	5 (3.5)	6 (4.2)
>3	70 (10.3)	2 (2.9)	3 (4.3)	6 (8.6)	5 (7.1)	6 (8.6)
Working lifetime (years)						
0–5	104 (15.3)	3 (2.9)	5 (4.8)	12 (11.5)	7 (6.7)	9 (8.7)
6–10	218 (32.2)	8 (3.7)	8 (3.7)	23 (10.6)	9 (4.1)	16 (7.3)
11–20	317 (46.8)	7 (2.2)	6 (1.9)	34 (10.7)	14 (4.4)	18 (5.7)
>20	39 (5.8)	2 (5.1)	0 (0.0)	7 (17.9)	1 (2.6)	1 (2.6)
Mask using						
No	497 (73.3)	18 (3.6)	16 (3.2)	62 (12.5)	26 (5.2)	37 (7.4)
Yes	181 (26.7)	2 (2.9)	3 (1.7)	14 (7.7)	5 (2.8)	7 (3.9)

a: *p* < 0.05; b: *p*_trend_ < 0.05.

**Table 3:** Influence factors of lung ventilation function among greenhouse workers.

	Variables in the Equation	B	Wald	*p*	OR (95% CI)
VC% predicted	Number of greenhouses owned		7.383	0.061	
	Number of greenhouses owned (1)	−1.244	5.130	0.024	0.288 (0.098, 0.846)
	Number of greenhouses owned (2)	−1.509	3.759	0.053	0.221 (0.048, 1.017)
	Number of greenhouses owned (3)	−0.791	1.019	0.313	0.453 (0.098, 2.106)
	Mask using	−1.208	2.576	0.108	0.299 (0.068, 1.306)
FVC% predicted	Age	0.868	2.872	0.090	2.383 (0.873, 6.505)
	Number of greenhouses owned		6.727	0.081	
	Number of greenhouses owned (1)	−1.142	4.199	0.040	0.319 (0.107, 0.951)
	Number of greenhouses owned (2)	−1.882	3.138	0.076	0.152 (0.019, 1.222)
	Number of greenhouses owned (3)	−0.046	0.004	0.947	0.955 (0.249, 3.668)
FEV_1_% predicted	Gender	−0.596	5.672	0.017	0.551 (0.338, 0.900)
	Age	0.862	11.163	0.001	2.368 (1.428, 3.927)
	BMI		5.385	0.068	
	BMI (1)	−0.948	4.295	0.038	0.388 (0.158, 0.950)
	BMI (2)	−1.066	5.319	0.021	0.345 (0.139, 0.852)
FEV_1_/FVC	-				
Lung ventilation function	Mask using	−0.693	2.700	0.100	0.500 (0.219, 1.143)

(1), (2) and (3) were the dummy variable contrast coding for logistic regression which were showed as Table A3.

**Table 4:** The mean values and SDs of small airway function indicators among greenhouse workers.

Variables	N (%)	MEF_50_% Predicted	MEF_25_% Predicted	MMEF% Predicted
Total	678 (100)	69.63 ± 24.95	54.04 ± 24.94	66.81 ± 24.53
Gender				
Male	291 (42.9)	69.44 ± 25.42	54.74 ± 25.77	67.05 ± 24.84
Female	387 (57.1)	69.77 ± 24.62	53.51 ± 24.31	66.63 ± 24.33
Age (years)				
≤47	365 (53.8)	71.33 ± 25.13	55.63 ± 26.04	68.84 ± 24.59 a
>47	313 (46.2)	67.66 ± 24.63	52.17 ± 23.49	64.45 ± 24.28
BMI				
<18.5	31 (4.6)	64.38 ± 25.74	53.07 ± 26.86	60.42 ± 2750
18.5–23.9	336 (49.6)	70.54 ± 25.57	53.75 ± 24.84	67.54 ± 24.31
>23.9	311 (45.9)	69.18 ± 24.19	54.43 ± 24.92	66.67 ± 24.45
Education				
Primary school and below	268 (39.5)	71.42 ± 24.91 a,b	53.43 ± 23.36	68.01 ± 24.10 b
Middle school	319 (47.1)	69.88 ± 25.26	55.05 ± 26.02	67.33 ± 25.08
High school and above	91 (13.4)	63.52 ± 23.25	52.26 ± 25.65	61.47 ± 23.39
Smoking				
Never-smokers	454 (67)	69.61 ± 25.20	53.30 ± 24.28	66.44 ± 24.58
Ever-smokers	224 (33)	69.68 ± 24.50	55.53 ± 26.22	67.57 ± 24.48
Number of greenhouses owned				
1	184 (27.1)	69.92 ± 24.03	55.67 ± 24.43	67.60 ± 24.54
2	281 (41.4)	69.28 ± 24.80	51.98 ± 24.16	65.69 ± 24.25
3	143 (21.1)	71.49 ± 26.52	57.49 ± 28.11	69.54 ± 26.01
>3	70 (10.3)	66.50 ± 24.81	50.94 ± 21.56	63.67 ± 22.26
Working lifetime (years)				
0–5	104 (15.3)	68.96 ± 23.64	53.62 ± 23.98	66.17 ± 25.31
6–10	218 (32.2)	68.19 ± 24.66	53.24 ± 25.14	65.72 ± 24.68
11–20	317 (46.8)	69.93 ± 25.40	53.93 ± 25.09	66.96 ± 24.24
>20	39 (5.8)	77.03 ± 25.87	60.45 ± 25.06	73.45 ± 23.83
Mask using				
No	497 (73.3)	69.18 ± 24.26	53.79 ± 24.92	66.44 ± 24.35
Yes	181 (26.7)	70.89 ± 26.52	54.69 ± 25.04	67.83 ± 25.05

a: *p* < 0.05; b: *p*_trend_ < 0.05.

**Table 5:** Positive rates of abnormal small airway function among greenhouse workers.

Variables	N (%)	MEF_50_% Predicted <65%	MEF_25_% Predicted <65%	MMEF% Predicted <65%	Abnormal Small Airway Function
Total	678 (100)	305 (45.0)	489 (72.1)	320 (47.2)	335 (49.4)
Gender					
Male	291 (42.9)	133 (45.7)	206 (70.8)	139 (47.8)	145 (49.8)
Female	387 (57.1)	172 (44.4)	283 (73.1)	181 (46.8)	190 (49.1)
Age (years)					
≤47	365 (53.8)	154 (42.2)	258 (70.7)	157 (43.0)a	164 (44.9) a
>47	313 (46.2)	151 (48.2)	231 (73.8)	163 (52.1)	171 (54.6)
BMI					
<18.5	31 (4.6)	16 (51.6)	22 (71.0)	18 (58.1)	18 (58.1)
18.5–23.9	336 (49.6)	150 (44.6)	243 (72.3)	152 (45.2)	161 (47.9)
>23.9	311 (45.9)	139 (44.7)	224 (72.0)	150 (48.2)	156 (50.2)
Education					
Primary school and below	268 (39.5)	113 (42.2)	187 (69.8)	116 (43.3) b	124 (46.3)
Middle school	319 (47.1)	143 (44.8)	236 (74.0)	154 (48.3)	159 (49.8)
High school and above	91 (13.4)	49 (53.8)	66 (72.5)	50 (54.9)	52 (57.1)
Smoking					
Never-smokers	454 (67)	201 (44.3)	333 (73.3)	211 (46.5)	224 (49.3)
Ever-smokers	224 (33)	104 (46.4)	156 (69.6)	109 (48.7)	111 (49.6)
Number of greenhouses owned					
1	184 (27.1)	75 (40.8)	120 (65.2)	76 (41.3)	81 (44.0）
2	281 (41.4)	135 (48.0)	213 (75.8)	143 (50.9)	148 (52.7)
3	143 (21.1)	59 (41.3)	102 (71.3)	64 (44.8)	67 (46.9)
>3	70 (10.3)	36 (51.4)	54 (77.1)	37 (52.9)	39 (55.7)
Working lifetime (years)					
0–5	104 (15.3)	49 (47.1)	73 (70.2)	45 (43.3)	51 (49.0)
6–10	218 (32.2)	96 (44.0)	162 (74.3)	102 (46.8)	105 (48.2)
11–20	317 (46.8)	148 (46.7)	229 (72.2)	159 (50.2)	166 (52.4)
>20	39 (5.8)	12 (30.8)	25 (64.1)	14 (35.9)	13 (33.3)
Mask using					
No	497 (73.3)	222 (44.7)	361 (72.6)	232 (46.7)	241 (48.5)
Yes	181 (26.7)	83 (45.9)	128 (70.7)	88 (48.6)	94 (51.9)

a: *p* < 0.05; b: *p*_trend_ < 0.05.

**Table 6:** Influence factors of small airway function among greenhouse workers.

	Variables in The Equation	B	Wald	*p*	OR (95%CI)
MEF_50_% predicted	-				
MEF_25_% predicted	Number of greenhouses owned		7.113	0.068	
	Number of greenhouses owned (1)	0.513	6.074	0.014	1.671 (1.111, 2.513)
	Number of greenhouses owned (2)	0.283	1.375	0.241	1.327 (0.827, 2.129)
	Number of greenhouses owned (3)	0.588	3.291	0.070	1.800 (0.954, 3.397)
MMEF% predicted	Age	0.506	8.417	0.004	1.659 (1.178, 2.335)
	Education		6.870	0.032	
	Education (1)	−0.487	3.923	0.048	0.614 (0.379, 0.995)
	Education (2)	−0.059	0.055	0.815	0.943 (0.577, 1.542)
Small airway function	Age	0.562	9.959	0.002	1.754 (1.237, 2.485)
	Education		5.424	0.066	
	Education (1)	−0.446	3.202	0.074	0.640 (0.393, 1.043)
	Education (2)	−0.068	0.072	0.788	0.934 (0.568, 1.535)
	Working lifetime		6.334	0.096	
	Working lifetime (1)	0.015	0.004	0.952	1.015 (0.632, 1.629)
	Working lifetime (2)	0.137	0.353	0.552	1.146 (0.731, 1.798)
	Working lifetime (3)	−0.775	3.724	0.054	0.461 (0.210, 1.012)

(1), (2) and (3) were the dummy variable contrast coding for logistic regression which were showed as Table A3.

## Appendix A

**Table A1:** Equation of calculating individual predicted value of lung function indicators developed by European Respiratory Society (ERS) in 1993.

Indicators	Equations	
	Male	Female
VC	0.0466 × (Height × 100) − 0.024 × Age − 3.28	0.061 × (Height × 100) − 0.028 × Age − 4.65
FVC	0.0576 × (Height × 100) − 0.026 × Age − 4.34	0.0443 × (Height × 100) − 0.026 × Age − 2.89
FEV_1_	0.0395 × (Height × 100) − 0.025 × Age − 2.60	0.0430 × (Height × 100) − 0.029 × Age − 2.49
MEF_50_	0.0379 × (Height × 100) − 0.031 × Age − 0.35	0.0245 × (Height × 100) − 0.025 × Age + 1.16
MEF_25_	0.0261 × (Height × 100) − 0.026 × Age − 1.34	0.0105 × (Height × 100) − 0.025 × Age + 1.11
MMEF	0.0194 × (Height × 100) − 0.043 × Age + 2.70	0.0125 × (Height × 100) − 0.034 × Age + 2.92

**Table A2:** Variable assignment of independent variables of logistic regression analysis.

Variable	Assignment
Gender	0 = male, 1 = female
Age, years	1 = ≤47, 2 = >47
BMI	1 = <18.5, 2 = 18.5 − 23.9, 3 = <23.9
Education	1 = Primary school and below, 2 = Middle school, 3 = High school and above
Smoking	0 = never−smokers, 1 = ever−smokers
Working lifetime, years	1 = 0−5, 2 = 6−10, 3 = 11−20, 4 = >20
Number of greenhouses owned	1 = 1, 2 = 2, 3 = 3, 4 = >3
Mask using	0 = No, 1 = Yes

**Table A3:** Dummy variable contrast coding for logistic regression.

Variable		N	Coding		
			(1)	(2)	(3)
BMI	<18.5	31	0	0	-
	18.5–23.9	336	1	0	-
	>23.9	311	0	1	-
Education	Primary school and below	268	1	0	-
	Middle school	319	0	1	-
	High school and above	91	0	0	-
Working lifetime, years	0–5	104	0	0	0
	6–10	218	1	0	0
	11–20	317	0	1	0
	>20	39	0	0	1
Numbers of greenhouses owned	1	184	0	0	0
	2	281	1	0	0
	3	143	0	1	0
	>3	70	0	0	1

**Table A4:** Relationship between mask using and possible influence factors.

	Mask Using		*p* ^a^
	No	Yes	
BMI			0.172
<18.5	25	6	
18.5–23.9	236	100	
>23.9	236	75	
Education			0.967
Primary school and below	195	73	
Middle school	235	84	
High school and above	67	24	
Number of greenhouses owned			0.901
1	136	48	
2	202	79	
3	106	37	
>3	53	17	

^a^ *p* value was calculated with Chi-square test.
